# Chemical modulators of the innate immune response alter gypsy moth larval susceptibility to *Bacillus thuringiensis*

**DOI:** 10.1186/1471-2180-10-129

**Published:** 2010-04-27

**Authors:** Nichole A Broderick, Kenneth F Raffa, Jo Handelsman

**Affiliations:** 1 Department of Entomology, University of Wisconsin, Madison, WI 53706, USA; 2 Department of Bacteriology, University of Wisconsin, Madison, WI 53706, USA; 3 Department of Plant Pathology University of Wisconsin, Madison, WI 53706, USA; 4 Current address: Global Health Institute, EPFL, Lausanne, 1004, Switzerland; 5 Current address: Department of Molecular, Cellular and Developmental Biology, Yale University, New Haven, CT 06520, USA

## Abstract

**Background:**

The gut comprises an essential barrier that protects both invertebrate and vertebrate animals from invasion by microorganisms. Disruption of the balanced relationship between indigenous gut microbiota and their host can result in gut bacteria eliciting host responses similar to those caused by invasive pathogens. For example, ingestion of *Bacillus thuringiensis *by larvae of some species of susceptible Lepidoptera can result in normally benign enteric bacteria exerting pathogenic effects.

**Results:**

We explored the potential role of the insect immune response in mortality caused by *B. thuringiensis *in conjunction with gut bacteria. Two lines of evidence support such a role. First, ingestion of *B. thuringiensis *by gypsy moth larvae led to the depletion of their hemocytes. Second, pharmacological agents that are known to modulate innate immune responses of invertebrates and vertebrates altered larval mortality induced by *B. thuringiensis*. Specifically, Gram-negative peptidoglycan pre-treated with lysozyme accelerated *B. thuringiensis*-induced killing of larvae previously made less susceptible due to treatment with antibiotics. Conversely, several inhibitors of the innate immune response (eicosanoid inhibitors and antioxidants) increased the host's survival time following ingestion of *B. thuringiensis*.

**Conclusions:**

This study demonstrates that *B. thuringiensis *infection provokes changes in the cellular immune response of gypsy moth larvae. The effects of chemicals known to modulate the innate immune response of many invertebrates and vertebrates, including Lepidoptera, also indicate a role of this response in *B. thuringiensis *killing. Interactions among *B. thuringiensis *toxin, enteric bacteria, and aspects of the gypsy moth immune response may provide a novel model to decipher mechanisms of sepsis associated with bacteria of gut origin.

## Background

The gut epithelium and its associated microorganisms provide an important barrier that protects animals from the external environment. This barrier serves both to prevent invasion by potential pathogens and limit the elicitation of host responses to the resident microbiota [1,2]. Dysfunction of this barrier, which can occur as a result of alterations of the normal gut ecology, impairment of host immune defenses, or physical disruption of intestinal epithelia, may lead to pathological states [3-6].

To breach the gut barrier, many enteric pathogens have evolved specific strategies such as production of toxins that physically disrupt cells of the gut epithelium [7-11]. *B. thuringiensis *kills insects through the production of such toxins, designated insecticidal crystal proteins. Following ingestion of *B. thuringiensis *by susceptible larvae, these toxins initiate killing of insects through a multi-step process that includes the formation of pores and lysis of midgut epithelial cells [12-15]. Despite a detailed understanding of the mechanisms of toxin binding and disruption of the midgut epithelium, we know less about the subsequent events that cause larval mortality. Three mechanisms, which account for differences among host responses, have been suggested as the ultimate cause of larval death. The first, in which larvae die from toxin ingestion within hours or a day, is attributed to direct toxemia [13,16,17]. The second, in which prolonged feeding on *B. thuringiensis *leads to developmental arrest and eventual death is thought to occur by starvation [18-20]. The third, and most commonly cited mechanism is sepsis due to the growth of *B. thuringiensis *in the hemocoel following translocation of spores from the toxin-damaged gut into the hemolymph [12,13,21,22]. However, despite numerous reports of growth of *B. thuringiensis *in dead or moribund larvae [23-26], there is little evidence of *B. thuringiensis *proliferation in insect hemolymph prior to death. In addition, the proposed mechanism of death by *B. thuringiensis *bacteremia is not supported by the ability of cell-free preparations of toxin [12,17,27], direct injection of some activated toxins into the hemocoel [28], or transgenic plant tissue producing the toxin [29] to kill larvae without the *B. thuringiensis *bacterium itself.

Previously, we demonstrated that *B. thuringiensis *toxin had substantially reduced ability to kill gypsy moth and three other species of lepidopteran larvae that had been treated with antibiotics, and that ingestion of an enteric-derived bacterium significantly increased lethality of subsequent ingestion of *B. thuringiensis *[30,31]. We observed that the enteric bacterium, *Enterobacter *sp. NAB3, grew to high population densities *in vitro *in hemolymph extracted from live gypsy moth larvae, whereas *B. thuringiensis *was rapidly cleared, which is inconsistent with the model of *B. thuringiensis *bacteremia as a cause of larval death. However, these results did not distinguish between the possibilities that gut bacteria contribute to *B. thuringiensis*-induced lethality by bacteremia or by another mechanism.

There is increasing recognition that an important feature of gut microbiota of both invertebrates and vertebrates is their ability to shape and modulate the host immune response [32-36]. In certain circumstances this effect can become deleterious to the host. For instance, uncontrolled activation of the immune response by enteric bacteria leads to chronic infection and pathogenesis in both invertebrates and vertebrates [37-39]. Interestingly, some recent studies have also linked activation of the immune response of Lepidoptera to ingestion of non-lethal doses of *B. thuringiensis*. For example, ingestion of low doses of *B. thuringiensis *by *Galleria mellonella *larvae increased both oxidative stress levels in the gut [40] and the phagocytic activity of hemocytes [41]. In *Trichoplusia ni *larvae, exposure to *B. thuringiensis *reduced both the numbers of hemocytes and components of the humoral immune response (antimicrobial peptides and phenoloxidase activity) [42]. It remains unclear what effectors trigger this immune modulation, and the contribution of enteric bacteria to this response is not known. Modulation of the host immune response could be an indirect mechanism by which gut microbiota alter susceptibility to *B. thuringiensis*.

As an initial step to distinguish between a direct or host-mediated role of gut microbiota in larval death following the ingestion of *B. thuringiensis*, we examined the possible association between the host immune response and larval susceptibility to *B. thuringiensis*.

## Results

### Effects of intra-hemocoelic injection of *B. thuringiensis *and *Enterobacter *sp. NAB3 on larval hemolymph

**Figure 1:**
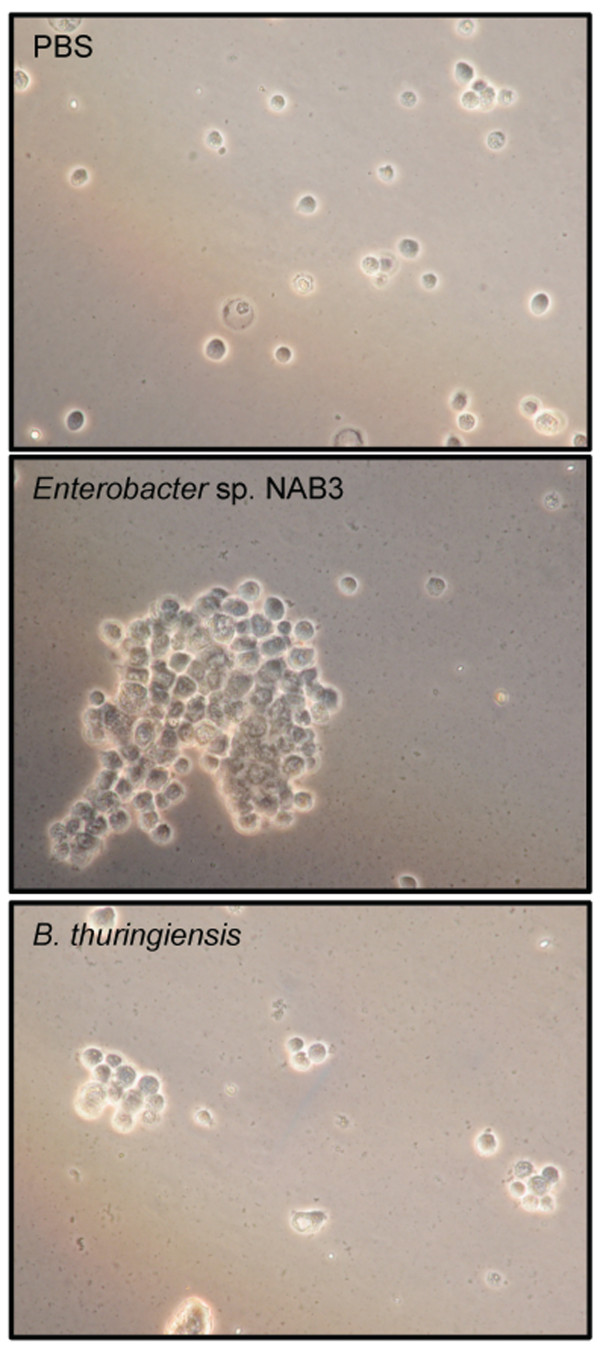
**Effect of intra-hemocoelic injection of *Enterobacter *sp. NAB3 or *B. thuringiensis *cells on hemocytes of gypsy moth larvae**. (a) 10 μl of PBS, (b) approximately 10^7 ^cells of *Enterobacter *sp. NAB3 or (c) *B. thuringiensis *(non-sporulated) were introduced into three separate cohorts of 4^th^-instar larvae (n = 10 each). Representative images of samples from each treatment are shown. To monitor the growth of injected bacteria, hemolymph samples were removed after 24 h and observed by light microscopy at 40×. Hemocytes from uninfected larvae were scattered randomly in the microscope field (a). In contrast, large aggregates of hemocytes were observed in samples from larvae injected with NAB3 (b) and smaller aggregates in samples from larvae injected with *B. thuringiensis *(c).

Injections of greater than 10^7 ^cells of an over-night culture of either *B. thuringiensis *or *Enterobacter *sp. NAB3 into the hemocoel of gypsy moth larvae led to a pronounced cellular and humoral immune response (Figure 1). In hemolymph sampled from larvae 24 h after injection of *Enterobacter *sp. NAB3, melanization of plasma and aggregation of hemocytes [43,44] were evident (Figure 1b). Hemocyte aggregation was also observed in hemolymph samples from larvae injected with *B. thuringiensis *(Figure 1c), though these aggregates appeared smaller than aggregates from larvae injected with *Enterobacter *sp. NAB3. Hemocyte aggregation was not observed in hemolymph sampled from control larvae (Figure 1a).

### Effects of ingestion of *B. thuringiensis *on larval hemolymph and mortality

**Figure 2:**
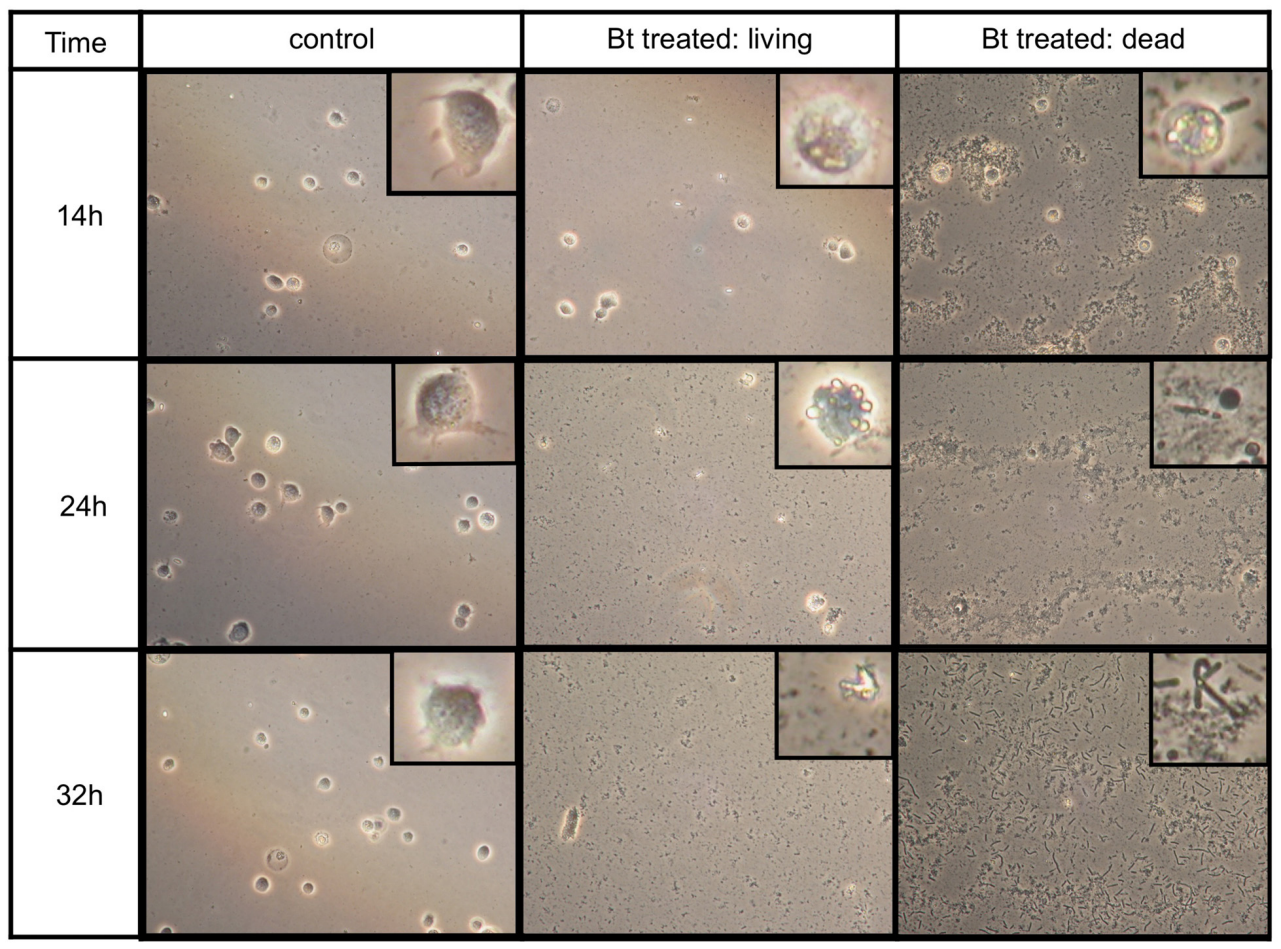
**Effect of ingestion of *B. thuringiensis *(DiPel 50 IU) on larval hemocytes**. Third-instar gypsy moth larvae were fed either distilled water or 50 IU of DiPel (n = 50). Hemolymph was sampled from a separate cohort of five larvae of each treatment at 0, 14, 24, and 32 h post-infection and examined by light microscopy (40×). Representative images are shown, including magnification of individual hemocytes (inset). No differences were observed among larvae from different treatments at 0 h (Additional file 1). Hemocytes from control larvae are adherent and emit pseudopodia (left panel). In contrast, hemocytes from larvae that ingested *B. thuringiensis *are non-adherent and contain inclusions (center panel). At the time points sampled, the majority of larvae fed *B. thuringiensis *were still alive. When present, dead larvae that had been fed *B. thuringiensis *were also sampled (right panel). In dead larvae, only a few abnormal hemocytes were detected and *B. thuringiensis *cells were present (right panel, insets). No mortality was observed in the controls that were not fed *B. thuringiensis*. Mortality values of control and *B. thuringiensis*-treated larvae corresponding to each time point are shown in Table 1.

We examined hemocytes and hemolymph in larvae containing enteric bacteria following oral ingestion of *B. thuringiensis *cells and toxin (Table 1). Microscopic examination of larval hemolymph revealed that the number of hemocytes declined following ingestion of *B. thuringiensis*. Defects in larval hemocytes were commonly observed within 14 h of ingestion of *B. thuringiensis*. This decrease in hemocyte abundance and appearance of defects occurred in advance of larval mortality. At 24 h post-ingestion of *B. thuringiensis*, larval mortality remained below 10%, even though 75% of samples contained fewer hemocytes and hemocytes with abnormalities (Table 1). Hemocytes from control larvae displayed no abnormalities and no larval mortality was observed (Figure 2; see also additional file 1). The hemolymph of uninfected larvae contained hemocytes, predominantly plasmatocytes and granulocytes, which displayed no abnormal characteristics. Moreover, these plasmatocytes retained the ability to adhere to a glass surface and form pseudopodia (Figure 2, left panel and insets). The plasma of control larvae remained free of debris or discoloration in samples taken over the course of the assay period, and no bacteria were observed over the course of the assay. In contrast, hemocytes from larvae fed *B. thuringiensis *were greatly reduced in number, lacked adhesive properties, and contained refractive inclusions and signs of membrane disruption (Figure 2, center panel and insets). As the number of hemocytes decreased, the plasma darkened and granular material or debris accumulated in samples (Figure 2, center and right panels). The loss of nearly all hemocytes corresponded with the onset of larval death (Table 1) and the appearance of *B. thuringiensis *cells in hemolymph samples (Figure 2, right panel and insets).

**Table 1:** Temporal sequence of effects of ingestion of a low dose of live cell formulation of *B. thuringiensis *(DiPel 10 IU) on condition of hemocytes and larval mortality in third-instar gypsy moth.

Time (h)	Larvae with hemocyte abnormalities^a ^(proportion)		Hemocyte rating^b^		Larval mortality (proportion)	
	**No treatment**	**Bt**	**No treatment**	**Bt**	**No treatment**	**Bt**

0	0.00	0.00	+++	+++	0.00	0.00
14	0.00	0.40	+++	++	0.00	0.02
24	0.00	0.75	+++	+	0.00	0.07
32	0.00	0.87	+++	+/-	0.00	0.15

^a ^n = 5 for each treatment.

^b ^Rating scale:

+++: hemocytes entire, adhesive properties

++: some hemocytes, inclusions present

+: very few hemocytes, ruptured cells

-: no hemocytes

### Effects of bacterial components capable of eliciting immune responses on larval susceptibility to *B. thuringiensis *toxin

**Figure 3:**
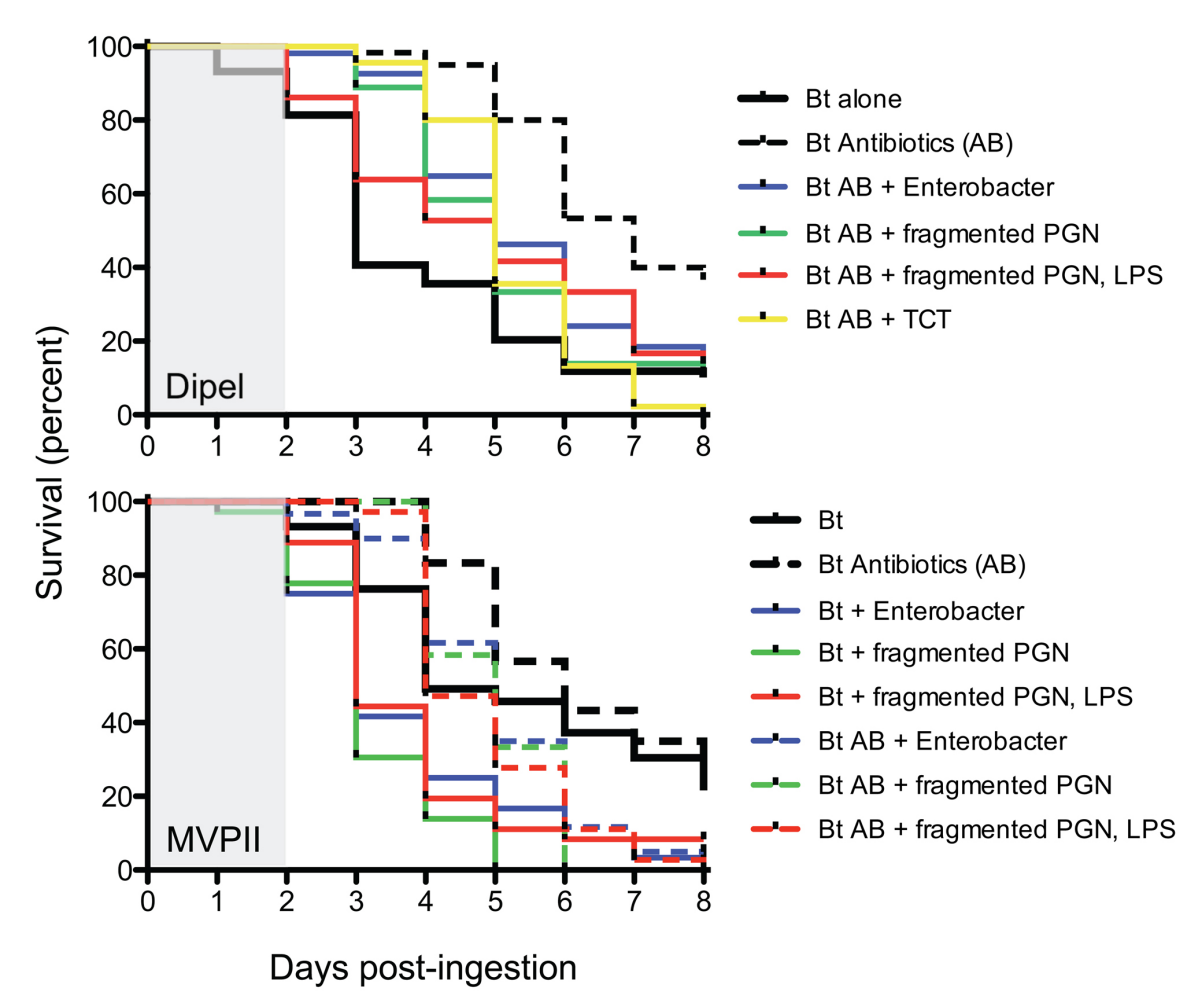
**Survival of third-instar gypsy moth larvae reared without enteric bacteria (antibiotics) or with enteric bacteria (no antibiotics) fed bacterial cell-derived compounds and *B. thuringiensis *(Bt)**. Two formulations of *B. thuringiensis*, DiPel 50 IU (upper) and MVPII 20 μg (lower), were assayed. All experimental treatments were provided on artificial diet without antibiotics, gray shading indicates days on which larvae received treatments. The effects of the compounds were assessed in comparison to *B. thuringiensis *toxin and significance of treatments was determined using the log-rank analysis of PROC LIFETEST (SAS 9.1, Table 2, Additional file 2). Treatments with a survival distribution function that differ significantly from *B. thuringiensis *toxin alone (p < 0.05) are shown; p-values of all treatments are presented in Table 2. Three independent cohorts of larvae were assayed. No mortality was observed when larvae were fed the compounds alone (Additional file 3).

Our observation that *B. thuringiensis *ingestion affected cellular immunity suggested the hypothesis that gut bacteria exert their effect on larval susceptibility to *B. thuringiensis *in part through stimulation of the host immune response. To determine whether bacterial cell components mediated *B. thuringiensis*-induced killing, we examined the effect of cell extracts known to trigger immune reactions in many invertebrate and vertebrate hosts, including Lepidoptera, [45-49] on gypsy moth susceptibility to *B. thuringiensis*. We examined the effect of commercial and purified lipopolysaccharide preparations and various peptidoglycan-derived compounds on larval mortality when co-administered with *B. thuringiensis*. As shown previously [30,31], rearing larvae on antibiotics reduced their susceptibility to *B. thuringiensis *(MVPII, p = 0.0202; Dipel, p < 0.0001, Table 2), and *Enterobacter *sp. NAB3 accelerated mortality of larvae fed *B. thuringiensis *plus antibiotics in assays using either *B. thuringiensis *Cry1Ac toxin (MVPII) or viable *B. thuringiensis *cells and toxins (DiPel) (Figure 3, Table 2; see also additional files 2 and 3). Feeding peptidoglycan from Gram-negative bacteria, solubilized by pre-treatment with lysozyme, in combination with *B. thuringiensis *reduced time to death of antibiotic-reared larvae (Figure 3, Table 2). Regardless of the *B. thuringiensis *formulation, the lysozyme-treated peptidoglycan accelerated mortality of antibiotic-treated larvae, and the effect of the lysozyme-treated peptidoglycan was not significantly different from *Enterobacter *sp. NAB3 (Figure 3). Restoration of killing by peptidoglycan was not affected by the addition of lipopolysaccharide to either *B. thuringiensis *formulation. There was no effect of either crude (peptidoglycan-contaminated [50]) or purified lipopolysaccharide or non-lysozyme treated-polymeric peptidoglycan on larval mortality with *B. thuringiensis *in antibiotic-treated larvae. Ingestion of monomeric peptidoglycan (tracheal cytotoxin) significantly accelerated mortality of larvae reared on antibiotics and treated with the live cell formulation of *B. thuringiensis *(DiPel, Figure 3, Table 2), but not with *B. thuringiensis *toxin alone (MVPII, Table 2).

**Table 2:** Effects of bacterial cell-derived immune elicitors on susceptibility of third-instar gypsy moth larvae reared without enteric bacteria (antibiotics) or with enteric bacteria (no antibiotics) to *B. thuringiensis *(Bt).

a) Bt cell preparation (DiPel, 50 IU)			
		**Reared without antibiotics**	**Reared with antibiotics**
**Rearing treatment**	**Elicitor added to *B. thuringiensis***	**Bt alone**	**Bt alone**

No Antibiotics	Bt alone	--	< 0.0001
No Antibiotics	*Enterobacter *sp. NAB3	0.6882	< 0.0001
Antibiotics	*Enterobacter *sp. NAB3	0.0956	< 0.0001
No Antibiotics	Crude lipopolysaccharide	0.8231	< 0.0001
Antibiotics	Crude lipopolysaccharide	0.0001	0.4942
No Antibiotics	Purified lipopolysaccharide	0.7268	< 0.0001
Antibiotics	Purified lipopolysaccharide	< 0.0001	0.5731
No Antibiotics	*Bacillus cereus *peptidoglycan	0.0582	0.0100
Antibiotics	*Bacillus cereus *peptidoglycan	0.0065	0.7331
No Antibiotics	*Vibrio fisheri *peptidoglycan	0.1092	< 0.0001
Antibiotics	*Vibrio fisheri *peptidoglycan	0.0010	0.1276
No Antibiotics	Tracheal cytotoxin	0.0539	< 0.0001
Antibiotics	Tracheal cytotoxin	0.4070	< 0.0001
No Antibiotics	Lysozyme-digested *V. fisheri *peptidoglycan	0.2622	< 0.0001
Antibiotics	Lysozyme-digested *V. fisheri *peptidoglycan	0.2356	< 0.0001
No Antibiotics	Lysozyme-digested *V. fisheri *peptidoglycan + purified lipopolysaccharide	0.1120	< 0.0001
Antibiotics	Lysozyme-digested *V. fisheri *peptidoglycan + purified lipopolysaccharide	0.2328	0.0002

**b) Bt Cry1Ac toxin (MVPII, 20 ug)**			

		**Reared without antibiotics**	**Reared with antibiotics**

**Rearing treatment**	**Elicitor added to *B. thuringiensis***	**Bt alone**	**Bt alone**

No Antibiotics	Bt alone	--	0.0202
No Antibiotics	*Enterobacter *sp. NAB3	< 0.0001	< 0.0001
Antibiotics	*Enterobacter *sp. NAB3	0.7182	0.0002
No Antibiotics	Crude lipopolysaccharide	0.6689	0.0919
Antibiotics	Crude lipopolysaccharide	0.0440	0.8517
No Antibiotics	Purified lipopolysaccharide	0.8138	0.0038
Antibiotics	Purified lipopolysaccharide	0.0456	0.5915
No Antibiotics	*Bacillus cereus *peptidoglycan	0.0651	< 0.0001
Antibiotics	*Bacillus cereus *peptidoglycan	0.0264	0.1951
No Antibiotics	*Vibrio fisheri *peptidoglycan	0.5111	0.0056
Antibiotics	*Vibrio fisheri *peptidoglycan	0.0196	0.8623
No Antibiotics	Tracheal cytotoxin	0.9977	0.0116
Antibiotics	Tracheal cytotoxin	0.0188	0.8914
No Antibiotics	Lysozyme-digested *V. fisheri *peptidoglycan	< 0.0001	< 0.0001
Antibiotics	Lysozyme-digested *V. fisheri *peptidoglycan	0.7613	0.0001
No Antibiotics	Lysozyme-digested *V. fisheri *peptidoglycan + purified lipopolysaccharide	0.0005	< 0.0001
Antibiotics	Lysozyme-digested *V. fisheri *peptidoglycan + purified lipopolysaccharide	0.5645	< 0.0001

Two formulations of *B. thuringiensis*, DiPel 50 IU (a) and MVPII 20 μg (b), were assayed. The significance (p-values) of the log-rank test comparing larval mortality of each experimental treatment group to Bt alone or Bt alone when reared with antibiotics is shown.

In the absence of antibiotics, larvae were highly susceptible to the live cell formulation of *B. thuringiensis *and the addition of bacterial compounds had no effect on larval survival rates (Table 2). However, the addition of *Enterobacter *sp. NAB3 and peptidoglycan fragments derived from bacteria accelerated mortality caused by *B. thuringiensis *toxin alone (MVPII, Figure 3). Neither preparation of lipopolysaccharide nor peptidoglycan that had not been treated with lysozyme affected mortality induced by the cell-free formulation of *B. thuringiensis *toxin (MVPII, Table 2).

### Effect of eicosanoid inhibitors and antioxidants on larval mortality associated with ingestion of *B. thuringiensis *toxin

To further test the hypothesis that larval susceptibility to *B. thuringiensis *toxin is modified by the host immune response to components of enteric bacteria, we fed larvae compounds previously demonstrated to inhibit the humoral and cellular immune responses of insects. Specifically, inhibitors of reactive oxygen and nitrogen species, phenoloxidase, and eicosanoid biosynthesis were fed to larvae to assess their effect on larval susceptibility to *B. thuringiensis *toxin. Five compounds, acetylsalicylic acid, indomethacin, glutathione, N-acetyl cysteine, and S-methyl-L-thiocitrulline, delayed mortality compared to larvae fed *B. thuringiensis *toxin alone. None of the compounds significantly affected final mortality and six had no effect on either the final mortality or survival time of larvae fed *B. thuringiensis *(Table 3).

**Table 3:** Effect of immune inhibitors on susceptibility of third-instar gypsy moth larvae reared without antibiotics to *B. thuringiensis *toxin (MVPII; 20 μg).

				Total Mortality (mean proportion ± SE)		
Compound added to *B. thuringiensis *toxin (MVPII)	Compound activity	Compound concentration	N	without *B. thuringiensis*	with *B. thuringiensis*	Significance (p-value) of rank analysis
*B. thuringiensis *toxin control			48	0.06 ± 0.02	0.92 ± 0.15 a	
Acetylsalicylic acid	Eicosanoid inhibitor (COX)	100 μg	36	0.00 ± 0.00	0.81 ± 0.16 ab	0.0396
Dexamethasone	Eicosanoid inhibitor (PLA_2_)	100 μg	24	0.00 ± 0.00	0.79 ± 0.19 ab	0.4519
Indomethacin	Eicosanoid inhibitor (COX)	10 μg	48	0.04 ± 0.04	0.83 ± 0.14 ab	0.0056
Esculetin	Eicosanoid inhibitor (LOX)	100 μg	24	0.00 ± 0.00	0.83 ± 0.18 ab	0.9757
Piroxicam	Eicosanoid inhibitor (COX)	100 μg	36	0.04 ± 0.02	0.94 ± 0.18 a	0.2417
Glutathione	Nitric oxide scavenger, phenoloxidase inhibitor	1.2 μg	36	0.02 ± 0.02	0.72 ± 0.14 ab	0.0154
N-acetyl cysteine	Reactive oxygen scavenger	100 mM	36	0.03 ± 0.01	0.86 ± 0.15 a	0.0286
Phenylthiourea	Nitric oxide scavenger, phenoloxidase inhibitor	75 mM	36	0.03 ± 0.03	0.81 ± 0.15 ab	0.3382
S-methyl-L-thiocitrulline	Nitric oxide scavenger	100 mM	36	0.03 ± 0.02	0.83 ± 0.15 ab	0.0245
Tannic acid	Phenoloxidase inhibitor	100 μg	24	0.00 ± 0.00	0.79 ± 0.19 ab	0.2740
S-nitroso-N-acetyl-l, l-penicillamine	Nitric oxide donor	100 mM	36	0.00 ± 0.00	0.94 ± 0.18 a	0.4409

The value N refers to the total number of larvae tested per treatment. There were no effects by these compounds without *B. thuringiensis*. Log-rank analysis was used to compare larval survival for each concentration of inhibitor, treatments with a p-value < 0.05 were considered significantly different from Bt toxin alone. Mean mortality values followed by the same letter do not differ significantly from each other.

**Figure 4:**
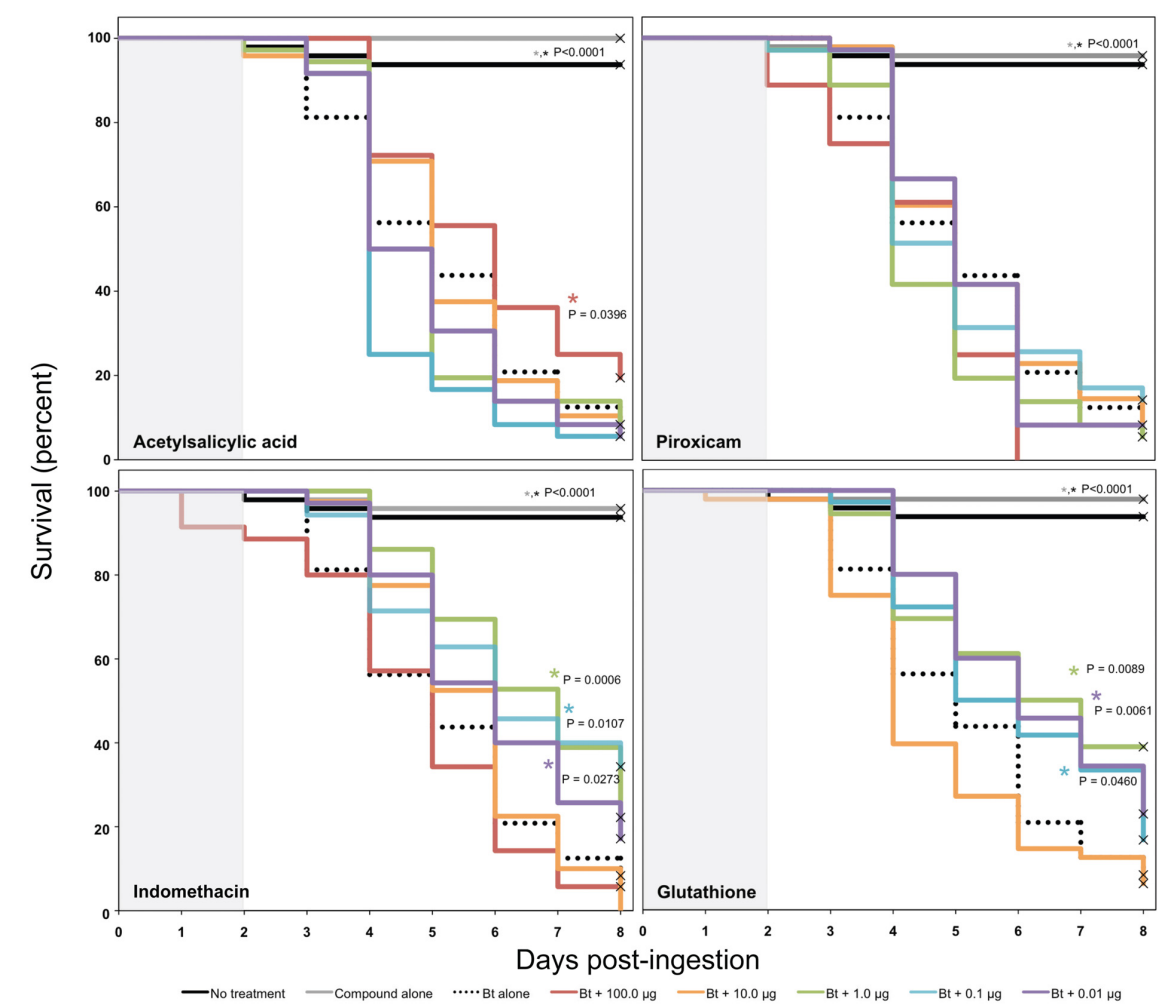
**Effect of antioxidants and eicosanoid inhibitors on survival of third-instar gypsy moth larvae following ingestion of *B. thuringiensis *toxin (Bt; MVPII 10 μg)**. Various concentrations of three COX inhibitors (acetylsalicylic acid, indomethacin, and piroxicam) and the antioxidant glutathione were fed to larvae in combination with 10 μg of the MVPII formulation of *B. thuringiensis *toxin. Larvae were reared with enteric bacteria (no antibiotics) and all treatments were provided on artificial diet without antibiotics; gray shading indicates days on which larvae received treatments. Three independent cohorts of larvae (n = 12-16 each) were assayed. No mortality was observed when larvae were fed the compounds alone (Additional file 4). The effect of the compounds was assessed by comparing survival to *B. thuringiensis *toxin alone using the log-rank anlaysis of PROC LIFETEST (SAS 9.1, Additional file 4). Treatments with a survival distribution function statistically different from *B. thuringiensis *toxin alone (p < 0.05) are indicated by *.

Dose-response assays with acetylsalicylic acid, glutathione, piroxicam, and indomethacin demonstrated complex relationships between inhibitor concentration and larval survival (Figure 4; see also additional file 4). Acetylsalicylic acid extended larval survival in the presence of *B. thuringiensis *toxin, but only at the high concentration (100 μg); the survival time of larvae treated with lower concentrations did not differ significantly from toxin alone. Most of the concentrations of indomethacin and glutathione that we tested increased larval survival time following ingestion of a lethal dose of *B. thuringiensis *toxin (Figure 4). Survival times of larvae treated with the highest concentrations of indomethacin and glutathione (100 μg and 12 μg, respectively) did not differ significantly from those treated with toxin alone.

## Discussion

Four lines of evidence indicate that the innate immune response is involved in *B. thuringiensis*-induced mortality of *L. dispar*. First, injections of *B. thuringiensis *and *Enterobacter *sp. NAB3 into the insect hemocoel were accompanied by melanization and hemocyte aggregation, both of which are indicators of an activated innate immune response. Second, as demonstrated here and reported by Ericsson et al. [42], depletion of hemocytes, the key actors of the cellular immune response of insects, was observed following *B. thuringiensis *ingestion in the absence of bacteremia. Third, fragments of peptidoglycan, an inducer of innate immunity, substituted for *Enterobacter *in accelerating killing of antibiotic-treated larvae with *B. thuringiensis*. Fourth, antioxidants and compounds that inhibit eicosanoid biosynthesis, and thereby suppress the innate immune response, delayed *B. thuringiensis*-induced mortality.

Based on these results, we propose the hypothesis that *B. thuringiensis *incites an overblown innate immune response, in cooperation with other factors, which in turn contributes to host death. This immune induction either requires the normal gut microbiota or is directly suppressed by antibiotic treatment, and is restored to antibiotic-treated larvae by addition of bacteria or immunostimulatory cell fragments. This model is derived, in part, from the mechanism of mammalian sepsis in which gut-derived microbiota serve as both sources of infectious bacteria and modulators of the innate immune system [51-54]. Germ-free mammals are less susceptible to sepsis, just as gypsy moth larvae lacking enteric bacteria are less susceptible to *B. thuringiensis *[53,55-57]. Further support for our model can be derived from recent work demonstrating that ingestion of non-pathogenic bacteria can induce the immune response of lepidopteran larvae [58]. This suggests that the microbiota are capable of altering the immune status of larvae without crossing the gut epithelium and could thus influence the host response to pathogenic bacteria. Additionally, Ericsson et al. [42] reported that reductions in the larval immune response following ingestion of a low dose of *B. thuringiensis *correlated with lower susceptibility to subsequent ingestion of *B. thuringiensis*. Taken together, these data provide support for the hypothesis that the host innate immune response contributes to pathogenesis and killing by *B. thuringiensis*.

We cannot rule out other factors that might co-vary with innate immunity. Many pharmaceutical inhibitors have non-specific effects on animals that may confound interpretation of the results [59-61]. While eicosanoids mediate various cellular reactions responsible for clearing bacterial infections from hemolymph circulation and are induced in Lepidoptera in response to bacterial challenge [62-64], they also have other physiological functions including ion transport and reproduction [60,65]. Thus, it is possible that the compounds we used have a direct effect on the health of the insect gut or affect another cellular process that, in turn, influences larval susceptibility to *B. thuringiensis*. Nevertheless, it is notable that we observed significantly delayed mortality with the antioxidant glutathione and in the presence of diverse compounds that suppress the synthesis of eicosanoids. The immune-suppressive compounds inhibit a variety of enzymes in eicosanoid biosynthesis, and all delay killing by *B. thuringiensis*, reducing the probability that the biological effects are due to a secondary activity of the pharmaceuticals. Moreover, peptidoglycan fragments, which induce the innate immune response, caused more rapid mortality in insects that had been treated with antibiotics.

Similarly, there is growing evidence that diverse classes of antibiotics, including the four used in this study, have immunomodulatory effects in addition to their antimicrobial activity [66]. While the immunomodulatory mechanisms of antibiotics are not fully understood, there is evidence that some directly reduce the host immune response, whereas others limit the release of immune-inducing bacterial components [67]. Further experiments are needed to fully differentiate the extents to which the reduction in susceptibility to *B. thuringiensis *when larvae are reared on antibiotics is due to the absence of gut bacteria or an immuno-suppressive effect of antibiotics. In the latter case, the re-introduction of bacteria, such as *Enterobacter *sp. NAB3, would likely stimulate the host immune response, and thus our current results do not permit us to separate these two possibilities. In either case, an immunomodulatory effect of antibiotics would further support a contribution of the host immune response in larval susceptibility to *B. thuringiensis*.

This is the third study, each with a different lepidopteran species, to report that ingestion of *B. thuringiensis *leads to alterations in hemocytes [41,42]. It remains unclear, however, whether the observed changes in hemocytes directly contribute to larval mortality or if they merely reflect changes in immune status. Interestingly, Ericsson et al. [42] reported that *T. ni *larvae resistant to *B. thuringiensis *had significantly fewer hemocytes than did susceptible larvae. Further experiments are needed to determine whether hemocytes are functionally required in susceptibility. Such experiments should include a comparison of the effect of ingestion of *B. thuringiensis *on hemocytes between larvae with and without enteric bacteria. In addition, while our work shows that immunogenic peptidoglycan fragments can restore *B. thuringiensis *susceptibility in larvae lacking gut bacteria, we do not know whether co-ingestion of peptidoglycan and *B. thuringiensis *leads to changes in hemocytes, nor have we identified the final immune effectors of *B. thuringiensis*-induced killing. However, the delayed mortality of larvae fed *B. thuringiensis *in combination with some antioxidants and eicosanoid inhibitors suggests that production of reactive oxygen species could be involved. Interestingly, hemocytes have been shown to be key regulators of the oxidative burst upon infection, particularly by promoting activation of the phenoloxidase cascade [68,69], which might be caused by hemocyte rupture [70,71].

The parallels between the progression of disease and mortality caused by *B. thuringiensis *with that in mammalian sepsis are noteworthy. Disease and death associated with mammalian sepsis are believed to be caused by uncontrolled host production of local immune mediators leading to local and systemic inflammatory responses [52,72,73]. Peptidoglycan induces the innate immune system of both invertebrates and vertebrates [45-49] and contributes to both sepsis and *B. thuringiensis*-induced killing in gypsy moth larvae. Eicosanoids and reactive oxygen and nitrogen species are critical in the innate immune response in mammals and treatments for sepsis often target these compounds [59,74-77]. In gypsy moth larvae, inhibitors of eicosanoid biosynthesis and antioxidants prevent or slow disease progress, suggesting a role of innate immunity.

There is increasing evidence that diseases of animals are frequently caused by multiple microbial species. These polymicrobial infections often include members of the indigenous microbiota and lead to complex interactions with the host immune system [74]. Using *Drosophila *as a model of cystic fibrosis, Sibley et al. [78] demonstrated that pathogenicity depends on synergy between *Pseudomonas aeruginosa *and members of the oropharyngeal microbiota. Even in the absence of infection changes in the gut immune response can lead to pathogenic states associated with an imbalance in composition of the gut microbiota [32].

Our results are consistent with the hypothesis that the effect of gut bacteria on host killing following ingestion of *B. thuringiensis *in antibiotic-treated larvae is mediated by the innate immune response. Further experiments, including direct monitoring of the immune response of larvae, are needed to identify the specific defense responses induced following ingestion of *B. thuringiensis *and the impact of antibiotic treatment and enteric bacteria on these events.

## Conclusion

We demonstrate that larvae fed *B. thuringiensis *die prior to observable growth of bacteria in the hemolymph. An immuno-stimulatory compound, fragments of Gram-negative peptidoglycan, confers *B. thuringiensis *toxin-induced killing in the absence of indigenous enteric bacteria. Conversely, inhibitors of the innate immune response delay mortality of larvae following ingestion of *B. thuringiensis *toxin. We propose the hypothesis that the resident gut bacteria in gypsy moth larvae induce an innate immune response that contributes to *B. thuringiensis *toxin-induced killing, suggesting a parallel with mammalian sepsis in which gut bacteria contribute to an overblown innate immune response that is ultimately lethal to the host.

## Methods

### Insects and rearing conditions

Eggs of *L. dispar *were obtained from USDA-APHIS. All eggs were surface sterilized with a solution of Tween 80 (polyoxyethylene sorbitan monooleate), bleach, and distilled water as previously described [79]. Larvae were reared in 15-mm Petri dishes on sterilized artificial diet (USDA, Hamden Formula) or sterilized artificial diet amended with antibiotics (500 mg/L of diet each penicillin, gentamicin, rifampicin, streptomycin). Larvae were reared under 16:8 (L:D) photoperiod at 25°C.

### Bacterial products and chemicals

Two commercial formulations of *B. thuringiensis*, alone and in combination with various bacterial products and compounds, were used in assays. The DiPel^® ^TD formulation consisted of cells, toxins (Cry1Aa, Cry1Ab, Cry1Ac, and Cry2A), and spores of *B. thuringiensis *subsp. *kurstaki *(Valent Biosciences, Libertyville, IL, USA). The MVPII formulation (DOW Agrosciences, San Diego, CA, USA) is comprised of Cry1Ac toxin encapsulated in NaCl-killed *Pseudomonas fluorescens*. *Enterobacter *sp. NAB3, a strain originally isolated from the midguts of gypsy moth larvae feeding on sterile artificial diet [80], was grown with shaking overnight in 1/2-strength tryptic soy broth at 28°C. The overnight culture was washed once and resuspended in 1× PBS (10^6 ^cells/μl) prior to use in assays.

Lysozyme and lipopolysaccharide from *Escherichia coli *0111:B4 were obtained from commercial sources (Sigma-Aldrich, St. Louis, MO). Peptidoglycan-free purified *E. coli *lipopolysaccharide, polymeric diaminopimelic (DAP)-type peptidoglycan from *Vibrio fisheri*, and monomeric DAP-type peptidoglycan, also called tracheal cytotoxin, from *Bordetella pertussis *were kindly provided by the laboratories of William E. Goldman and Margaret J. McFall-Ngai. Peptidoglycan from *Bacillus cereus *was provided by S. Brook Peterson [81]. The following chemicals were obtained from Sigma-Aldrich, St. Louis, MO: acetylsalicylic acid, dexamethasone, esculetin, glutathione, indomethacin, N-acetyl-cysteine, phenylthiourea, piroxicam, S-methyl-L-thiocitrulline, tannic acid, S-nitroso-N-acetyl-I, I-penicillamine.

### Intra-hemocoelic injections and hemolymph sampling

Fourth instar larvae were anesthetized by chilling on ice for 15 min, then surface sterilized with 95% ethanol (EtOH). Injections were performed with a 20-μl fixed-volume pipette and a snipped 200-μl pipette tip fitted with a 27-gauge needle. The syringe needle was inserted into the ventral abdomen between the first and second pair of prolegs, keeping the needle parallel to the body wall to avoid injuring the alimentary canal. Control larvae were injected with 10 μl of phosphate buffered saline (PBS). Experimental larvae were injected with 10 μl of a washed culture of *Enterobacter *sp. NAB3 or *B. thuringiensis *subsp. *kurstaki *adjusted to a concentration of 10^6 ^cells/μl. Larvae were maintained in 15 mm Petri plates by treatment group (n = 10) and provided with unamended sterile artificial diet for the duration of the assay. Hemolymph samples from larvae of each treatment were examined for bacteria 24 h after injection. Hemolymph was collected by piercing the last abdominal proleg with a 27-gauge needle and collecting the hemolymph drops with a 10-μl fixed-volume pipette. Approximately 10 μl of hemolymph was collected individually from five larvae for each treatment and diluted in PBS, 10 μl of which was spotted onto a plate of 1/10-strength tryptic soy agar, while the other 10 μl was placed on a glass slide for immediate microscopic observation.

### Temporal monitoring of hemolymph following ingestion of *B. thuringiensis *toxin

*B. thuringiensis *mortality assays were performed as previously described [30]. All assays were performed on newly molted third-instar larvae using sterile artificial diet without antibiotics. Either sterile water or 50 IU of DiPel was applied in a volume of 1 μl to a standard diet disk (3-mm diameter, 1-mm height) and fed to larvae. Hemolymph samples were collected as described above for microscopy from five control larvae and five *B. thuringiensis*-treated larvae at 14, 18, 24, and 32 h after treatment. Additionally, hemolymph samples from 5 larvae were examined at the commencement of treatment (0 h). Additionally, mortality was monitored in a parallel cohort of larvae for the duration of the assay.

### Feeding assays with immune elicitors

The effects of bacterial elicitors of the immune response of invertebrates and vertebrates on mortality following ingestion of *B. thuringiensis *were compared in larvae with indigenous gut bacteria (reared on unamended sterile diet) and axenic larvae (reared on diet amended with antibiotics) using two formulations of *B. thuringiensis*, MVPII and DiPel. All treatments were applied in 1-μl doses to a standard diet disk and fed to third-instar larvae on two consecutive days, at sample sizes shown in Table 2. All elicitors were tested alone to assess direct toxicity. Lysozyme-treated DAP-type peptidoglycan was prepared by incubating 5 mg/ml peptidoglycan in 1% lysozyme [5 mg/ml lysozyme in 0.1 M sodium acetate buffer (pH 5.0)] for 20 min, followed by heating the mixture at 95°C for 5 min to inactivate lysozyme.

### Feeding assays with eicosanoid inhibitors and antioxidants

The effects of eicosanoid inhibitors and antioxidants on mortality resulting from ingestion of the MVPII formulation of *B. thuringiensis *were assayed in larvae reared on unamended sterile artificial diet. Each compound was fed alone and in combination with MVPII for two days as described above and mortality was recorded daily for 9 days, at sample sizes indicated in Table 3. Subsequently, a dose-response for four of the inhibitors, acetylsalicylic acid, indomethacin, glutathione, and piroxicam, was established using the same protocol.

### Statistical analysis

Mean larval mortality and standard error were determined with data from either three or four replications of 10 to 12 larvae each using PROC MEANS [82]. Means were separated using Fisher's LSD at P = 0.05. The effect of bacterial elicitors or chemical inhibitors on time to death of *B. thuringiensis *treated larvae was analyzed using PROC LIFETEST [82]. Median survival times and their standard errors were obtained using the Kaplan-Meier estimation and rank analysis of PROC LIFETEST [82]. Survival curves of larvae fed *B. thuringiensis *toxin and various concentrations of acetylsalicylic acid, indomethacin, glutathione, and piroxicam were compared to *B. thuringiensis *toxin alone using the rank analysis of PROC LIFETEST [82].

## Authors' contributions

NAB performed all experiments. NAB and KFR performed the statistical analysis of the data. NAB, JH, and KFR conceived of and designed the study. NAB, JH and KFR analyzed the data and wrote the manuscript. All authors approve the final manuscript.

## Supplementary Material

Additional file 1 **Figure S1**. Effect of ingestion of *B. thuringiensis *(DiPel 50 IU) on larval hemocytes at t = 0 h. Click here for file

Additional file 2 **Table S1**. Summary of the log-rank statistics of survival of third-instar gypsy moth larvae following ingestion of *B. thuringiensis *toxin and various bacterial cell components. Click here for file

Additional file 3 **Table S2**. Larval mortality to bacterial cell-derived compounds in the absence of *B. thuringiensis*. Click here for file

Additional file 4 **Table S3**. Summary of the log-rank statistics of survival of third-instar gypsy moth larvae following ingestion of *B. thuringiensis *toxin and various concentrations of three COX inhibitors. Click here for file
