# Supplementary material for: Chemical modulators of the innate immune response alter gypsy moth larval susceptibility to Bacillus thuringiensis
Source: BMC Microbiol. 2010 Apr 27;10:129. doi: 10.1186/1471-2180-10-129 (PMC2873493; doi:10.1186/1471-2180-10-129)
Supplement: Additional file 1 — Figure S1. Effect of ingestion of B. thuringiensis (DiPel 50 IU) on larval hemocytes at t = 0 h. [file 1471-2180-10-129-S1.PDF]

Figure S1: Effect of ingestion of *B. thuringiensis* (DiPel 50 IU) on larval hemocytes. Third-instar larvae were fed either distilled water or 50 IU of DiPel (n=50). Hemolymph was sampled from five larvae of each treatment 10 min after they were observed ingesting artificial diet treated with *B. thuringiensis* (considered 0 h post-infection) and examined by light microscopy (40X). Representative images are shown. As there was no mortality at 0 h, no dead larvae could be sampled. No differences were observed among larvae from different treatments at 0 h.

Hours post-infection	control	Bt treated living	Bt treated dead
0 h	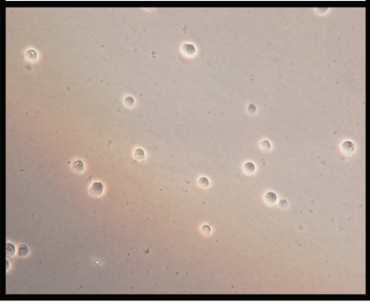	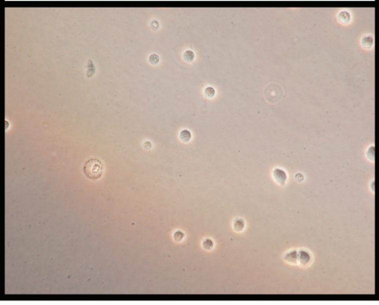	NA
